# Allergy in Cancer Care: Antineoplastic Therapy-Induced Hypersensitivity Reactions

**DOI:** 10.3390/ijms24043886

**Published:** 2023-02-15

**Authors:** Bianca Galateanu, Alexandra Ioana Pușcașu, Simona Andreea Tircol, Bogdan Cosmin Tanase, Ariana Hudita, Carolina Negrei, George-Traian-Alexandru Burcea-Dragomiroiu, Lucian Negreanu, Ileana Adela Vacaroiu, Octav Ginghină

**Affiliations:** 1 Department of Biochemistry and Molecular Biology, Faculty of Biology, University of Bucharest, 050095 Bucharest, Romania; 2 Department of Drug Control, Faculty of Pharmacy, “Carol Davila” University of Medicine and Pharmacy, 020021 Bucharest, Romania; 3 Fundeni Clinical Institute, 022328 Bucharest, Romania; 4 Department of Internal Medicine (Gastroenterology), Faculty of Medicine, “Carol Davila” University of Medicine and Pharmacy, 050474 Bucharest, Romania; 5 Institute of Oncology Prof. Dr. A. Trestioreanu Bucharest, 022328 Bucharest, Romania; 6 Departament of Toxicology, Faculty of Pharmacy, “Carol Davila” University of Medicine and Pharmacy, 020021 Bucharest, Romania; 7 “Sf. Ioan” Emergency Clinical Hospital, 042122 Bucharest, Romania; 8 Department of Nephrology, “Carol Davila” University of Medicine and Pharmacy, 050474 Bucharest, Romania

**Keywords:** oncology, infusion reaction, antineoplastic therapy, chemotherapy, cancer

## Abstract

As the backbone of oncological treatments, systemic chemotherapy is still one of the main pawns in cancer care, alone or in combination with newer targeted agents. All chemotherapy agents can be associated with a type of adverse event called an infusion reaction, which can be characterized as unpredictable, non-dose related, and unexplained by the cytotoxic profile of the drug. For some of these events, a certain immunological mechanism can be identified by blood or skin testing. In this case, we can speak of true hypersensitivity reactions that occur as a response to an antigen/allergen. The current work summarizes the main antineoplastic therapy agents and their susceptibility to induce hypersensitivity reactions and also includes a review of clinical presentation, diagnostic methods in hypersensitivity reactions, and perspectives to overcome these negative events in the treatment of patients suffering from various types of cancer.

## 1. Introduction

**Figure 1:**
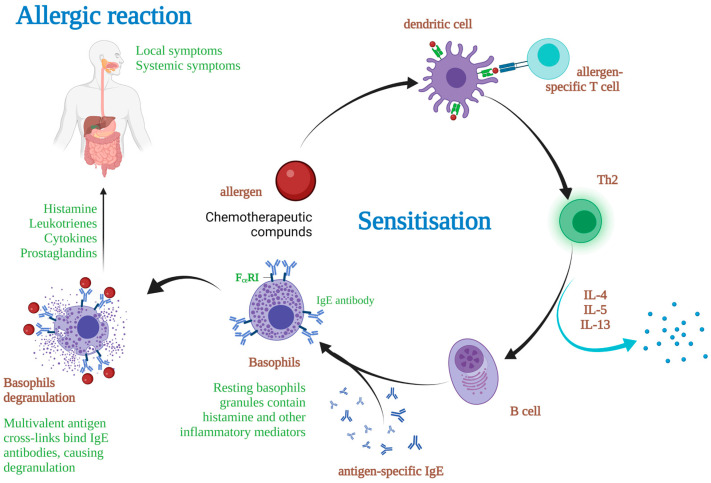
Basophilic mediated allergic reaction mechanism.

As widely accepted at present, in addition to their benefits, medicines can also be accompanied by side effects and adverse reactions, of which some can be detrimental to therapies or even life-threatening. In some cases, these effects are enabled or enhanced by certain individual-specific hypersensitivity. Among other manifestations, adverse reactions to drugs resulting from excessive sensitivity may include anaphylaxis. In most cases, this is an IgE-mediated response, caused by the activation of mast cells by IgE, triggering synthesis and release by mast cells of multiple chemical mediators among which notably histamine and certain metabolites of arachidonic acid (e.g., proteoglycans as, for instance, heparin, prostaglandins and leukotrienes, proteases), all formed beforehand and with known potent inflammation-mediating properties (Figure 1) [1]. The specific mechanism of mast cell activation consists of several stages, the first of which involves degranulation and more intense development of leukotrienes (LTC/D4 and LTB4) and prostaglandins (PGD2) resulting from arachidonic acid in the membrane, all contributing to clinical manifestations. Several developments follow in the mast cell activation process, including the release of other factors of which the most effective ones are chemokines and cytokines (mainly TNFa and IL-6), helping to engage additional effector cells such as eosinophils, basophils, and Th2 cells. These have an important impact on allergic response immunopathology, an increase of IgE level in the serum, and finally allergic sensitization [2].

As the backbone of oncological treatments, systemic chemotherapy is still one of the main pawns in cancer care, alone or in combination with newer targeted agents. Practically all chemotherapy agents can be associated with a type of adverse event called infusion reactions, which can be characterized as unpredictable, non-dose related, and unexplained by the cytotoxic profile of the drug—type B adverse reactions according to the European Medicines Agency (EMA) [3]. They usually appear during or shortly after drug administration, resolving after the interruption of infusion. Most of them are mild; however, more severe clinical pictures can be described. For some of these events, a certain immunological mechanism can be identified by blood or skin testing. In this case, we can speak of true hypersensitivity reactions (HSR) that occur as a response to an antigen/allergen and can be further categorized in 4 types according to Gell and Coombs classification (Table 1).

Type I is IgE-mediated and consists of symptoms such as urticaria, angioedema, bronchospasm, and anaphylaxis. Type II involves the cytolytic antibodies (IgG or IgM), inducing hemolytic anemia. In type III the antigen-antibody immune complexes cause vasculitis, while cell-mediated sensitized T lymphocytes determine type IV HSR, leading to contact dermatitis [4].

Since the terminology is not yet clearly defined, the terms “infusion” and “hypersensitivity” reaction are frequently used interchangeably; however, we should keep in mind that not all infusion reactions have an immunological basis. The non-immune-mediated infusion reactions, without a defined allergic mechanism, can present themselves as pseudo-anaphylaxis, with a symptomatology pattern similar to IgE-mediated HSR (for example in the context of cytokine release syndrome with subsequent mast cell degranulation) and idiosyncratic adverse events (unpredictable, uncommon) [3,5].

Infusion reactions to systemic chemotherapy appear more frequently with the use of agents such as platinum analogs, taxanes, asparaginase, pegylated liposomal doxorubicin, epipodophyllotoxins, bleomycin, cytarabine, and ixabepilone. For some of these drugs, an underlying allergic mechanism can be demonstrated; however, HSR with features of anaphylaxis has been reported for almost all of them, depending on individual susceptibility [3,6,7].

## 2. Antineoplastic Therapy Involved in Hypersensitivity Reactions

In this section, the most relevant classes of antineoplastic molecules (Table 2) inducing important hypersensitivity reactions are discussed.

### 2.1. Platinum Compounds

Used for a broad range of malignancies, platinum analogs have made a major contribution to systemic treatments. With a similar platinum core, cisplatin, carboplatin, and oxaliplatin are the main representatives of this class. Nedaplatin (Japan), heptaplatin (Korea) and lobaplatin (China) are three other therapeutic agents from this category, marketed in single countries. The different complexities of the leaving group and carrier ligand offer distinctive pharmacological proprieties and anticancer activity profiles to each agent. Their mechanism of action involves covalently binding to purinic DNA bases, creating cross-link strands, and inhibiting the normal function of nucleic acids with consecutive apoptosis [19].

In terms of HSR, platinum compounds are a class associated with the high potentiality to cause such events, carboplatin being the main one responsible, followed by oxaliplatin and cisplatin. A true allergic mechanism consistent with type I IgE-mediated HSRs can be established for most of the platinum analogs reactions [6,20]. Different variables have been investigated in various studies with different designs as risk factors for developing hypersensitivity events to these analogs. The most consistent risk factor seems to be prior exposure to these therapies, with the rate of HSR increasing after the administration of several cycles [20]. A summary of other potential risk factors for carboplatin, oxaliplatin, and cisplatin is presented in Table 3.

Details regarding the HSR associated with the main representatives of the class are provided in the following paragraphs.

#### 2.1.1. Cisplatin

The first member of its class, cisplatin, also known as cisplatinum or cis-diamminedichloroplatinum (II) (CDDP) is used in different combination regimens for the management of various types of solid carcinomas (such as ovarian, lung, bladder, head and neck, upper-GI tumors), as well as sarcomas, lymphomas, and germ cell neoplasias. Its toxic profile makes it more cautiously prescribed, being associated with nephrotoxicity, ototoxicity, neurotoxicity, and high emetogenic potential [19]. In early studies, the incidence of reported HSR to cisplatin was between 5 and 14%; however, premedication with glucocorticoids as part of the antiemetic regimen may have lowered over time the percentage of people who experienced infusion reactions [7]. However, the incidence may be further influenced by concomitant radiation, due to associated increased tumor necrosis and cytokine release [32]. The clinical picture of an HSR to cisplatin can vary from mild reactions to anaphylaxis, with pruritus, urticaria, dyspnea, and hypotension [20,32]. The symptoms frequently appear shortly (10–30 min) after the beginning of the intravenous infusion. The chance of HSR occurrence increases with the number of cycles, and is higher after the 6th cycle. Although there are not so many studies investigating the exact immunological mechanism behind cisplatin HSR, an IgE-mediated process is suggested by the presence of positive skin tests, described especially in studies of cross-reactivity with carboplatin and oxaliplatin and identification of patients who can be safely rechallenged with platinum salts [32,33,34].

#### 2.1.2. Carboplatin

With a better toxicity profile than its parent compound cisplatin, carboplatin (cis-diamminecyclobutanedicarboxylato platinum II) proved to be effective, especially in advanced or metastatic ovarian carcinoma, as well as other tumors such as cervix, lung, testicular, progressive diffuse large B-cell lymphoma, but with a lower efficacy on the germ cells malignancies [19]. Of all the platinum agents, carboplatin has the highest rate of associated HSRs. The incidence of all types of infusion reactions to carboplatin rises with repeated courses of administration, being up to 40% after at least seven doses [6]. One study reported a risk of up to 100% of developing an HSR to carboplatin in the third-line setting, after multiple exposures [35]. Re-treatment with carboplatin is consistently reported in the literature as the main risk factor for developing HSR [6,20,22]. The clinical features of HSR to carboplatin are highly variable, from mild to severe, but most of them resemble anaphylaxis, with symptoms such as pruritus, flushing, urticaria, facial swelling, chest tightness, dyspnea, cough, abdominal cramping, and hypotension [21,22,26,36,37]. Chest pain, followed by unresponsive cardiac arrest was also reported [26]. Reactions to carboplatin are usually acute, during infusion or shortly after, but can also be delayed, occurring hours or days after infusion [36]. The presence of maculopapular rashes approximately six days after exposure can be classified as a delayed reaction, associated with a higher risk of developing severe clinical phenomena in subsequent cycles [38]. Since a common carboplatin regimen also includes paclitaxel, which is usually infused before the platinum agent, some HSRs to carboplatin may be in fact due to the taxane. However, in contrast to platinum agents, taxanes-associated HSRs do not come in the context of previous exposures and occur typically during the first or second cycle of administration, with more atypical symptoms. These features can help in the accuracy of differential diagnosis [20]. Regarding the immunological basis, there is a large amount of evidence supporting a type I, Ig E mediated, allergic reaction to carboplatin, with positive skin tests as well as the presence of IgE in peripheral blood of patients with such events [22,39].

#### 2.1.3. Oxaliplatin

Among newer platinum compounds, oxaliplatin is particularly effective in the management of colorectal cancers, in combination with 5-fluorouracil (5FU), both in adjuvant and metastatic settings. The therapeutic action of this analog also extends to other gastrointestinal malignancies such as esophageal, gastric, and pancreatic cancers [19]. Parallel to the increased incidence of colorectal cancer cases, especially in young adults, the expanded usage of oxaliplatin in the clinical setting results in higher reported HSR to this platinum compound, up to 25% [6,40]. The HSRs appear usually during or shortly after administration and include a broad spectrum of manifestations, from cutaneous symptoms (rash, flushing, pruritus) to respiratory (bronchospasm, dyspnea), gastrointestinal (nausea, vomiting), and even cardiovascular (hypo, hypertension) phenomena [22]. Abdominal pain, chill, fever, diaphoresis, and other unspecific symptoms can also be present. Delayed reactions are rare, with few cases presented in the literature [41]. According to a large report, most of the reactions appeared after five cycles of administration, with the majority of the patients having only mild symptoms such as local erythema or pruritus. However, an elevated percentage (37%) of patients experienced severe reactions compatible with anaphylaxis, characterized by alterations in blood pressure, bronchospasm, chest tightness, diffuse erythroderma, or facial swelling [42]. Anaphylactic reactions to oxaliplatin are also described in various other case reports and series [28,43,44,45]. The immunologic mechanism involved in oxaliplatin-related HSR has been extensively studied. Rapid onset of manifestations during or after infusion, usually after multiple exposures along with positive prick tests and IgE detection in patients with reactions to oxaliplatin argues in favor of type I HSR, which should respond to desensitization [17,23,28,46]. Other immune-mediated reactions, which resemble a type II HSR, including immunologic thrombocytopenia, immune hemolytic anemia, Evans syndrome, and drug-induced thrombotic microangiopathy have also been reported for oxaliplatin [28,42,45,47,48,49,50]. Some of these cases resulted in exitus [49,50]. Moreover, based on clinical presentation and biomarkers, a group recently proposed a classification of oxaliplatin hypersensitivity reactions in four different endotypes, respectively: type 1, cytokine release, mixed, and either. Therefore, a cytokine release, a mast cell independent mechanism was suggested for the atypical clinical presentations that include fever, chills, rigors, headaches, and chest pain, in patients who also associated high levels of serum TNF and IL1 [29]. Notably, the HSR to oxaliplatin can be induced by concomitant administration of other drugs, such as leucovorin (within the FOLFOX regimen). Although there are fewer cases reported in the literature, leucovorin can also be responsible for HSR, therefore presumed oxaliplatin reactions can be an infusion reaction to racemic calcium folinate [51,52]. With the help of skin prick tests or drug provocation tests, allergy experts can establish the nature of the problematic drug and propose solutions such as the replacement of infusional 5FU and leucovorin with capecitabine.

#### 2.1.4. Cross-Reactivity among Platinum Agents

Cross-reactivity between platinum agents has been reported in different studies, with a percentage of up to 45% for oxaliplatin and carboplatin, based on clinical symptomatology as well as skin testing [17,33,34]. It is suggested that patients exposed to oxaliplatin can also be unable to tolerate the other platinum salts, so further testing should be done before switching to another analog [17]. In particular, recent studies reported that the administration of cisplatin after allergic reactions to carboplatin and oxaliplatin appears to be safe [33,34]. However, anaphylaxis to the parent platinum compound was still described after a previous similar reaction to carboplatin in smaller NSCLC case reports, therefore it is still unclear to which extent we can use cisplatin in this context [53]. The newer compound, nedaplatin was also investigated as a substitute for patients experiencing HSR to carboplatin. A recent study confirmed the safety profile and also efficacy of nedaplatin used in this context, with only one patient experiencing an HSR to this newer analog [54].

### 2.2. Taxanes

Taxanes are complex alkaloid esters that provide antitumor activity in a broad spectrum of solid malignancies, having as main representatives: paclitaxel, docetaxel, and the more recent addition cabazitaxel. Their mechanism of action consists of interfering in the dynamics and thread milling of the mitotic spindle, and stabilizing the microtubules, with subsequent activation of the pathways related to apoptosis [19]. Taxanes are highly susceptible to causing HSRs. However, in contrast to platinum agents, taxanes-induced HSR have usually different features and occurs during first or second exposure, with more atypical clinical symptoms thought to be anaphylactoid rather than IgE-mediated [55]. This reaction is probably due to a direct release of mast cell mediators such as histamine and tryptase, in the context of the non-immune effects of the drugs or their excipients (cremophor and polysorbate). Nevertheless, new data have emerged and currently more theories are being explored, including an IgE-mediated mechanism [20,55]. Details concerning HSRs induced by each member of the taxane class are discussed in the following paragraphs.

#### 2.2.1. Paclitaxel

The main indications of paclitaxel include management of advanced ovarian cancer, advanced breast cancer in the neoadjuvant as well as adjuvant setting, first-line treatment of small-cell lung cancer as well as subsequent therapy for AIDS-related Kaposi sarcoma. However, its usage expands to other malignancies, such as gastric, head and neck, bladder, cervical and esophagus cancers [19]. Before the extensive implementation of preventive methods, almost 30% of patients receiving paclitaxel were with HSR. This percentage diminished to less than 5% with the help of appropriate premedication and prolongation of the drug infusion time [6,55]. Paclitaxel is known to cause both standard infusion reactions as well as pseudo-anaphylaxis, either due to the chemotherapeutic component or the solvent used. When present, the clinical symptoms of a paclitaxel HSR outline a picture that includes erythematous rash, urticaria, dyspnea, bronchospasm, and hypotension, although some cases with hypertension have been described. Patients can also complain of gastrointestinal symptoms, back and chest pain [7,20]. Symptoms usually occur within a maximum of 15 min from the beginning of the infusion and in more than 90% of the cases during the first two cycles [7,20,55]. This pattern of immediate reaction has been considered to be more suggestive of a direct mast cell degranulation process, a mechanism that has been explored by several studies. The solvent and emulsifying agent CremophorEl, formulated with paclitaxel, have been proven to induce direct complement activation with consecutive mastocyte and basophils anaphylatoxins induced activation. Direct chemotherapy-induced degranulation with histamine release has also been proposed [55,56]. However, recent studies also raised the possibility of some HSR being IgE-mediated [7,55]. Although less frequently reported in the literature, delayed paclitaxel HSR have also been described, the majority of them presenting as cutaneous modifications such as rash, urticaria, and even angioedema [7,55]. They are considered to be an indicator of an immediate HSR to the next exposure. One case report described a female patient who died of anaphylactic shock following paclitaxel infusion, after presenting lip swelling and urticaria 10 days after the previous cycle [57]. Another potential delayed reaction to paclitaxel includes interstitial pneumonitis, which is associated with the need for mechanical ventilation and high mortality rates [58]. A form of subcutaneous lupus erythematosus, characterized by the presence of specific antibodies and skin eruption has also been described in conjunction with paclitaxel administration [59]. More, there is described in the literature at least one case of paclitaxel-induced Steven Jonson syndrome [60].

#### 2.2.2. Docetaxel

Docetaxel proved its efficacy in locally advanced or metastatic breast cancer, locally advanced or metastatic NSCLC, metastatic castration sensitive or resistant prostate cancer, advanced gastric adenocarcinoma including esophagogastric junction tumors, and inoperable locally advanced squamous cell of head and neck cancers [19]. In early phase II studies of Docetaxel, up to 30% of the patients experienced acute HSR, but later with the use of appropriate premedication with antihistamines and corticosteroids, the percentage remained under 2% [58,61,62]. Same as with paclitaxel, the reactions appear fast, in the first 10–15 min after infusion and usually in the first or second cycle of administration. The clinical picture of this event, as described by a study presenting 102 NSCLC patients with HSR to docetaxel, includes facial flushing, chest discomfort, back pain, increased heart rate, erythematous rash, cardiovascular alterations such as important hypotension and urticarial [58]. Similar to paclitaxel, non-immediate hypersensitivity reactions, such as immunological interstitial pneumonitis and a particular form of subcutaneous lupus erythematosus have also been described [63,64]. Concerning the immediate docetaxel-induced HSR, most of the works indicate a non-allergic nature of these events [65]. It has been demonstrated in vitro that both docetaxel and its solvent and emulsifying agent polysorbate 80 and polysorbate alone can be responsible for complement activation and subsequent mast cell activation as well as peroxide-induced degranulation [20,55,66,67]. This theory does not apply to delayed reactions, the non-immediate hypersensitivity events being mediated by a cellular immunological response [68].

#### 2.2.3. Cabazitaxel

Cabazitaxel is one of the newest additions to the taxane class, being used for the management of castration-resistant metastatic prostate cancer. Opposed to the other taxanes, cabazitaxel has a low affinity for P glycoprotein, a drug exporter, supporting its use for docetaxel-resistant prostate tumors [55]. HSRs to cabazitaxel were reported in phase I and II studies [69,70]. Clinical manifestations have the same pattern of developing as the other taxanes, a few minutes after infusion, in the first two cycles. Given the rapid occurrence of the reactions, they are most likely caused by the non-immune-mediated effects of the emulsifier Polysorbate 80 [69,70,71]. The symptomatology might include rash, erythema, bronchospasm, and sometimes hypotension. As with docetaxel, the main cause for the reactions is thought to be the emulsifier Polysorbate 80 [71]. Nevertheless, in a phase III study where 378 patients were assigned to receive cabazitaxel after docetaxel progression, none experienced HSR to the drug, so overall a clear incidence for these events is still unknown [72].

#### 2.2.4. Nab-Paclitaxel

With activity in breast cancer, NSCLC, and alongside gemcitabine in pancreatic cancer, Nab-Paclitaxel is a special formulation of paclitaxel contained in particles of human albumin and not Cremophor El. As a result, this chemotherapy drug is associated with fewer HSRs and does not require a special premedication regimen, strengthening the proposed idea that reactions to simple paclitaxel are usually due to the formulation. Severe infusion reactions were rarely described, mainly including dyspnea, desaturation, and back pain [73,74].

#### 2.2.5. Cross-Reactivity between Taxanes

It appears that it is a high level of cross-reactivity between paclitaxel and docetaxel, which may vary among populations, so caution may be used before substituting one taxane for another [55,75]. A larger study showed that half of the patients who presented an HSR to paclitaxel also developed a reaction when the taxane was switched to docetaxel, for a cross-sensitivity rate of 50% (7 out of 14 patients). Since docetaxel and paclitaxel have different solvents, polysorbate 80 and cremophor L, respectively, the authors suggested that given the high percentage of cross-reactivity, it is more likely that the taxane moiety itself is responsible for the allergic reactions [75]. However, a more successful use was described in several case reports of breast and ovarian cancer for nab-paclitaxel, which was safely administered after docetaxel or paclitaxel HSR [76,77,78].

### 2.3. Disruption of Protein Synthesis

Based on the observation that leukemia cells need extracellular asparagine to grow, a hydrolyzing agent for the nonessential amino acid was developed for the treatment of hematological cancers in the form of L-asparaginase. As part of a multidrug scheme for acute lymphoblastic leukemia, asparaginase is known to be associated with HSR, both mild and severe reactions. Currently, there are five formulations of asparaginase available, the non-pegylated forms being considered more immunogenic [79,80,81]. Data shows that approximately 10–30% of subjects receiving *E. coli*-derived asparaginase and 3–37% receiving *Erwinia* asparaginase experience clinical symptoms after infusion [79]. They may occur from the first administration, in this case being unlikely associated with an immune-mediated mechanism. However, the risk increases after multiple cycles, being the highest during the consolidation and reinduction phases [6]. The HSR picture can include dyspnea, pruritus, bronchospasm, skin rash, urticaria, and sometimes hypotension, phenomena that usually appears in the first minutes of infusion, but can also occur later, after a few hours. However, less than 10% of the cases are considered severe [6,7]. Such events can also appear following intramuscular or subcutaneous forms of administration, but with less frequency and include pain, tenderness, swelling, and erythema at the injection site [6,7,79]. Pegylated formulations are more associated with delayed HSR [6,7,79]. The immunological basis of the asparaginase-related HSR has been proven, with antidrug antibodies present in the peripheral blood of patients with such adverse events [79]. Switching to asparaginase with a different immunologic profile might be a solution to HSR, especially for *E. coli*-derived products, with over 90% of the patients being able to complete the treatment [7,79].

### 2.4. Bleomycin

Derived from the fungus *Streptomyces verticillus*, bleomycin has strong antitumor activity, especially in germinal cell tumors, gestational trophoblastic disease, and Hodgkin non-Hodgkin lymphoma. Its mechanism of action consists of DNA cleavage [19]. Most of the infusion reactions to bleomycin do not seem to have a clear IgE-mediated background, this agent is associated with more idiosyncratic reactions such as hyperpyrexia, hypersensitivity pneumonitis, or chest pain [7]. An old study showed no evidence of histamine release, or hypotension after drug administration, supporting the idea of a non-IgE-mediated mechanism [82]. However, we managed to identify at least one case report of anaphylaxis to bleomycin in a patient with Hodgkin’s lymphoma and a case of fatal angioedema 48 h after drug administration [83,84]. Not related to cancer, another case report described an intraoperative allergic reaction following the injection of bleomycin for sclerotherapy [85]. Nevertheless, it also appears that bleomycin has the potential to aggravate atopic dermatitis and stimulates airway hyperactivity and inflammation in mouse models, therefore more work is needed to elucidate the underlying reaction mechanism [86].

### 2.5. Topoisomerase II Inhibitors

#### 2.5.1. Epipodophyllotoxins

Demethylepipodophyllotoxin derivatives, etoposide (VP 16), and teniposide (VM-26) exert their antitumor activity by inhibiting the activity of the enzymes topoisomerase II. Etoposide is mainly used in the treatment of small-cell lung cancer and testicular tumors, while teniposide is more usually administered in the setting of refractory leukemia [19]. In terms of HSRs to this etoposide, a clinical picture with anaphylaxis can develop following intravenous administration, with hypotension, bronchospasm, chest tightness, and urticaria, usually appearing after multiple exposures [6,87]. However, the incidence seems to be low, around 1–3% [6]. The oral formulation of etoposide is not associated with any HSR, suggesting the fact that intravenous infusion reactions are probably due to polysorbate-80, the same solvent used for docetaxel that causes complement activation and subsequent mast cell direct degranulation [6,87]. Another form of VP16, etoposide phosphate is a water-soluble prodrug with no trace of polysorbate 80 that has successfully proven a good substitute agent after etoposide HSR [87,88]. Despite being extremely rare, more recently, severe anaphylactic-like reactions to this newer formulation have also been described [89]. Regarding teniposide, an older comprehensive analysis showed an incidence of 6.5% of HSR to VM-26, with a higher percentage in children with neuroblastoma and brain tumors, compared to hematologic malignancies [90]. The underlying mechanism seems to be a dose-dependent one, with direct degranulation of basophils following infusion.

#### 2.5.2. Anthracyclines and Other Related Agents

Originally antibiotics, anthracyclines are currently used in oncological clinical practice, in both solid and hematological malignancies. Their biological mechanism consists of topoisomerase II inhibition and intercalating with the DNA. Doxorubicin and its less cardiotoxic analog epirubicin are mainly used in solid tumors, especially breast cancers, sarcomas, and lymphomas, while daunorubicin and its analog idarubicin are part of chemotherapy protocols in the setting of acute leukemia. With the help of liposomal technology, a newer anthracycline formulation named pegylated liposomal doxorubicin is also used in breast cancer cases with increased cardiological risk and in platinum-resistant ovarian cancers [19]. Anthracyclines (including intravesical-administered doxorubicin) are rarely associated with hypersensitivity reactions. The clinical characteristics of a doxorubicin-related infusion reaction may include rash, dyspnea, headache, back pain, chills, and sometimes hypotension [91]. More frequently, anthracyclines can cause a local flare reaction near the site of drug administration place with erythema or pruritus, but without progressing to a generalized event [7,91]. Nevertheless, with formulations such as pegylated liposomal doxorubicin or liposomal daunorubicin, the incidence of HSRs is around 9–14% [7]. Without premedication, up to 45% of patients can develop such adverse events [92]. However, the pseudo-anaphylaxis seem to be caused by the surface component of the liposome and not the chemotherapy agent itself, generating a direct complement activation [93]. True IgE is not proven to be involved [91,94].

### 2.6. Alkylating Agents—Cyclophosphamide/Ifosfamide

Used in the oncology field for the treatment of ovarian cancers, breast carcinomas, lymphomas, leukemia, neuroblastoma, and retinoblastomas, cyclophosphamide acts as an alkylating agent of the nitrogen mustard type, with a potent anticancer and immunosuppressive action. Anaphylaxis is rarely associated with cyclophosphamide infusion, with only a few cases described in the literature [95,96,97]. However, when present, an HSR to this cancer agent tends to develop up to 16 h after infusion, the proposed mechanism is an IgE-mediated reaction to the two main metabolites phosphoramide mustard, and acrolein [96].

A synthetic analog of cyclophosphamide, Ifosfamide is another agent with alkylating activity used in a wide variety of neoplasia such as gynecological and lung cancers, as well as in sarcomas and non-Hodgkin’s lymphomas [19]. Ifosfamide is also infrequently associated with HSR. However, when present, most ifosfamide-related reactions are considered to be due to MESNA, an agent used for preventing hemorrhagic cystitis, a very important side effect of both cyclophosphamide and ifosfamide administration [98,99,100].

### 2.7. Antimetabolites—Pyrimidine Analogues

The pyrimidine analog, also known as arabinosylcytosine (ARA-C), cytarabine is an antineoplastic antimetabolite agent mainly used to treat hematological malignancies, especially acute non-lymphocytic leukemia, acute lymphocytic leukemia and blast phase of chronic myelocytic leukemia [101]. Anaphylaxis with symptoms such as urticaria, angioedema, or hypotension is rarely reported for cytarabine. Cases of delayed hypersensitivity are also extremely rare, with cutaneous lesions with pruritus, rash, and erythematous maculae being described three days after infusion [102]. Most of the infusion reactions associated with this agent consist of a more flu-like syndrome, with myalgia, arthralgia, conjunctivitis, and skin modifications. This “cytarabine syndrome” appears in one-third of the patients and it is thought to be associated with a cytokine release mechanism and can usually be prevented with appropriate premedication [101].

### 2.8. Monoclonal Antibodies

Similar to cytotoxic chemotherapy, monoclonal antibodies used in cancer management can also cause infusion reactions [103,104,105]. Most of them usually appear during the first or second drug infusion, between 30 min and 24 h after initiation of perfusion, with clinical characteristics such as fever, chills, back or abdominal pain, skin rashes, nausea, but also cardiovascular alterations, and dyspnea. A recent study conducted on 104 patients proposed four different pathophysiological backgrounds for Monoclonal induced infusion reactions, including cytokine-mediated, mastocytes and basophils mediated (type I like), T cell and macrophages mediated (type 4 like) and not least mixed reactions [103]. The highest incidence of infusion reactions is reported with the use of avelumab, rituximab, daratumumab, and alemtuzumab, with more than 50% of patients developing clinical symptoms, and in slightly lower percentages with trastuzumab (40%) and cetuximab (20%) [104,105]. While most of the infusion reactions associated with these agents coincide with the cytokine-mediated pattern, Type 1 allergic reactions have also been described with rituximab, trastuzumab, and cetuximab, these agents are known to cause both types of events [104].

#### 2.8.1. Rituximab

A chimeric monoclonal antibody CD20 targeted, Rituximab is used in cancer care for the management of CD20 positive B-cell Non-Hodgkin’s Lymphoma (NHL) and Chronic Lymphocytic Leukemia (CLL), but also non-oncological settings for diseases such as pemphigus Vulgaris or rheumatoid arthritis. More than half of the patients have rituximab infusion-related reactions appearing after 30 min of intravenous administration, with symptoms suggestive of a cytokine-mediated pattern such as fever, chills, back pain, sweat, or rhinitis [104,106,107]. The use of subcutaneous rituximab can also induce similar systemic symptoms [104,106]. It is thought that secondary to the interaction of the monoclonal antibody to the T cell surface marker CD20, a variety of cytokines are released by the targeted cell, with even potentially life-threatening manifestations such as pulmonary infiltration or acute respiratory distress syndrome. It seems that fractioning rituximab and using premedication can significantly lower the rates of this kind of reaction [103,105]. However, in less frequent cases, a clinical picture of anaphylaxis with bronchospasm, pruritus, urticaria, and hypotension has also been described, with positive skin tests or IgE specific to rituximab [103,104,105,106]. The information provided in the literature is too thin to establish a relationship between the type of hypersensitivity reactions and the disease for which rituximab is used. However, it might be interesting to investigate whether there is an association between the different doses and schedules of administration of rituximab in oncological and non-oncological settings and the class of HSR. Type IV Gell and Coombs HSRs can also occur in the cancer setting, with Stevens-Johnson syndrome being reported in at least one case of B cell lymphoma [108], while type III HSR is usually reported in non-oncological cases.

#### 2.8.2. Trastuzumab

Targeting the extracellular domain of the human epidermal growth factor receptor (HER2), trastuzumab is used in different combinations for the treatment of HER2-positive breast cancer, in adjuvant and palliative settings, and also for metastatic gastric and gastroesophageal tumors. This monoclonal antibody is generally associated with infusion reactions during first intravenous administration, most of them being thought to be non-IgE-mediated, with symptoms comprising of chills, fever, nausea, rash, headache, abdominal pain and rhinitis that seems to appear in over 40% of women with HER2 positive disease [109,110]. Moreover, in a retrospective study on 197 breast cancer patients receiving trastuzumab, only three experienced infusion reactions after more than one administration, suggesting a predominant cytokine-dependent mechanism [109]. Although in the summary of product characteristics, it is suggested that the infusion reactions can occur up to 6 h after drug intravenous administration, a more recent paper proved that these events can only occur during the 90 min of infusion [111]. These types of reactions can be managed by temporizing the infusion or slowing the rate of administration, further occurrence of similar symptomatology does not usually appear in subsequent cycles [109,111]. Despite being extremely rare, clinical pictures consisting of anaphylaxis are also described with the use of intravenous trastuzumab [111]. Urticaria, angioedema, hypotension, and severe dyspnea can signal a type I allergic reaction to the administration of this monoclonal antibody. In such cases, protocols of desensitization can be used to further pursue this type of therapy, when there are no other better therapeutic alternatives [112]. According to a recent research paper regarding grade III hypersensitivity events for the combination trastuzumab pertuzumab, the incidence seems to be very low for the intravenous form and with no reported severe reactions for the subcutaneous formulation [111]. Compared to trastuzumab, other HER2 targeted agents such as Ado-trastuzumab emtansine or fam-trastuzumab deruxtecan are rarely associated with infusion reactions, most of them being mild with classical characteristics such as fever, chills, shortness of breath [113].

#### 2.8.3. Cetuximab

Causing infusion reactions in up to 25% of patients, cetuximab is a human/mouse chimeric antibody that targets the epidermal growth factor receptor EGFR, used in the management of metastatic KRAS wild-type metastatic colorectal cancer and head and neck tumors [110]. Although being known of causing a cytokine release pattern of reactions, a more clearly defined IgE-mediated mechanism was described for some of the more severe reactions [110,114]. The serum analysis of patients with cetuximab-induced severe infusion reactions showed the presence of IgE antibodies directed to galactose-alpha-1,3-galactose, an oligosaccharide presents on the cetuximab mouse-derived heavy chain, unraveling a type I allergic reaction [114]. Therefore, we can easily explain the lack of cross-reactivity between cetuximab and panitumumab, the latest being a fully human-derived monoclonal antibody, lacking galactose-alpha-1,3-galactose [115]. It is also suggested that a history of atopy can act as a risk factor for anaphylaxis in cetuximab-treated patients [116]. In terms of prevention, a more recent Korean nationwide study on 64 patients concluded that the determination of IgE antibodies to cetuximab or galactose-α-1,3-galactose can accurately predict the future development of anaphylaxis in patients that will receive the drug and can be used in clinical practice [117].

At the end of this section, we summarize in Table 4 the reactions reported for each drug:

## 3. Clinical Presentation and Diagnostic Methods in HSRs

### 3.1. Clinical Presentation of HSRs

HSR to chemotherapeutic drugs has been encountered in most therapeutic agents. Their clinical presentation has a great degree of variability, from the immunopathogenic mechanisms that determine them, to the time of occurrence and their degree of severity [4,6]. These HSR-associated signs and symptoms target different organs and systems. The skin can be affected by rash, pruritus, urticaria, angioedema, palmar erythema, and facial flushing. Ocular itching, hyperemia, tearing, and periorbital edema are possible signs of ophthalmological involvement. The nasal mucosa can be affected by itching, rhinorrhea, congestion, and sneezing. The oral cavity and lips are involved through itching or tingling, metallic taste, and angioedema, while the larynx is targeted through symptoms such as the sense of swelling, dysphonia, hoarseness, dysphagia, and stridor. The involvement of the respiratory tract induces bronchospasm. Abdominal pain, nausea, or diarrhea are typical gastrointestinal symptoms. Gynecological involvement can manifest as vaginal itching, uterine cramps/bleeding, and incontinence. Neurologically, patients can experience, anxiety and a sense of impending doom, an altered mental status, and seizures. The cardiovascular system is targeted through variations in blood pressure and heart rate. These reactions can evolve in severity, manifesting as chest pain, angina pectoris, anaphylaxis, and, rarely, death. The aforementioned severe forms of clinical presentation tend to occur during the chemotherapeutic infusion, while mild or moderate manifestations can develop at any moment, from the onset of the chemotherapeutic administration to a 24–72 h time span post-infusion [4,6].

### 3.2. Diagnostic Methods

#### 3.2.1. Skin Tests (STs)

Skin tests (STs) are tools employed, alongside medical history and physical examination, in diagnosing IgE-mediated disorders, namely allergic rhinitis, asthma, and anaphylaxis induced by aeroallergens, foods, insect venoms, or certain drugs. The two categories of STs used by clinicians are percutaneous testing (prick or puncture) and intracutaneous testing (intradermal). The first type requires a needle puncturing the upper layers of the skin through a drop of allergen extract placed previously on the surface of the tegument, followed by a gentle elevation of the epidermis. This technique can be performed with the use of several devices. On the other hand, intradermal testing implies injecting a small quantity of allergen into the dermis. The physiopathology of the reaction involves mast cell secretion of preformed histamine, followed by smooth-muscle contraction and increased vascular permeability, leading to the development of a wheal. Concomitantly, inflammatory mediators generate a neural reflex, with secondary vasodilation and erythema (flare). Taking into account the higher specificity and finer correlation with clinical sensitivity, percutaneous testing is the first method employed in IgE detection. However, intracutaneous testing is associated with a more elevated degree of sensitivity. When evaluating the probability of a systemic reaction, intradermal testing has been proven to pose a slightly higher risk in comparison to prick tests, thus supporting the primary usage of the percutaneous technique, followed, in the event of a negative result and a remaining clinical suspicion, by intracutaneous testing. STs can be performed on the back or the volar surface of the arm, the latter enabling the patient to observe and sense the emergence of pruritus. The increasing number of sensitized patients (with IgE antibodies and secondary positive STs) that have no associated symptomatology, has imposed the existence of clinical manifestations to establish the diagnosis of an allergic reaction [118].

Skin prick tests and intradermal tests can be valuable tools in evaluating IgE-mediated chemotherapy reactions, with proven utility for platinum compounds and taxanes. In addition to the allergic response generated by the active substance, one must also consider the effect of emulsifying agents included in the drug formulas. Moreover, premedication drugs such as steroids, and serotonin 5HT3 receptor antagonists can also cause HSR. Furthermore, results of STs for the emulsifying agents included in the drug formulas have, also, not been provided. The current review focuses on allergic reactions induced by active compounds of the chemotherapeutic agents [6]. 

Since the 1990s, carboplatin STs have been used in evaluating and managing HSR, with differences in practice from institution to institution [20]. A single intradermal test has a negative predictive value (NPV) ranging from 81% to 92% [119], while an exact estimation of the positive predictive value (PPV) can be difficult to obtain, considering the ethical constraints of challenging patients after a positive ST result. However, the study conducted by Markman et al. reveals that 6 out of 7 patients with a positive ST suffered an HSR with standard infusion, resulting in a PPV of 86% [120]. Regarding oxaliplatin, there are conflicting opinions, as a recent study failed to find an association between ST and allergic reactions [29], while the research conducted by Alvarez-Cuesta et al. provided sensitivity and specificity values of 57.5% and 91.7%, respectively [121]. There are limited data concerning cisplatin ST [23]. Additional studies with rigorous designs are needed to properly evaluate the predictive values of ST. Carboplatin and oxaliplatin testing are useful tools in stratifying the risk of patients with a history of HSR. The likelihood of experiencing HSR during desensitization is greater among ST-positive patients, compared to the ones with a negative result [121,122]. The issue of cross-reactivity to other platinum-containing drugs needs to be evaluated, as there are literature reports of cisplatin and oxaliplatin severe reactions in patients with a personal history of carboplatin allergy [123,124]. However, a recent study conducted by Pasteur et al., evaluating 155 patients, has proven an extremely low degree of cross-reactivity between cisplatin and other platinum agents [34]. Thus, in the absence of other chemotherapeutic options of a different class, a negative ST can guide the selection of an alternative platinum agent [6]. 

Although ST has not been a routine testing tool for taxanes, as they were considered to have a non-IgE-mediated allergic mechanism, there is a subset of patients with IgE-mediated HSR based on a history of yew tree pollen sensitization. Paclitaxel and, more rarely, docetaxel have been proven to determine positive ST, unlike cabazitaxel or nab-paclitaxel [20].

ST-positive reactions associated with HSR have been reported in other chemotherapeutic drug classes such as cyclophosphamide, procarbazine, gemcitabine, methotrexate, and L-asparaginase, but further studies are needed to establish their diagnostic and predictive values [6].

The non-irritating concentrations of most chemotherapeutic agents mentioned above for STs are presented in Table 5.

#### 3.2.2. Drug Provocation Tests (DPTs)

DPTs consist of a controlled administration of a certain drug to diagnose an immune- or non-immune-mediated HSR. It is considered to be the final point and the “gold standard” investigation to accurately recognize drug hypersensitivity in the case of negative or unavailable previous diagnostic evaluations. The advantage of DPT is recreating the symptoms and other adverse clinical reactions irrespective of the underlying mechanism. The disadvantages consist of the lack of standardized protocols (protocol is chosen based on patients’ reports about the reaction suffered), potential danger, the possibility of a false positive or false negative result, subjective symptoms and lack of objective biomarkers, risk of resensitization, and the need for intervention of experienced personnel. The DPT contraindications are uncontrolled asthma/underlying chronic disease, pregnancy, use of beta-blockers, personal history of heart disease (may impend the use of adrenaline), vasculitis syndromes, bullous exanthemas, acute generalized exanthematous pustulosis, drug-induced autoimmune disease/hypersensitivity syndromes, personal history of organ-specific disorders, severe anaphylaxis. This investigation is employed solely if other standard tests fail to show meaningful results and after carefully evaluating the risk-benefit ratio. The elements that impact the decision and the protocol for a DPT are the chronology of the main clinical reaction (immediate vs. non-immediate), the severity of the reaction (anaphylaxis vs. mild reactions), the age of the patient (child vs. adult) and the existence of an intensive care unit in the center performing the investigation [125,126]. Regarding the antineoplastic therapy regimens, DPT helps rule out chemotherapy-associated hypersensitivity, in evaluating patients receiving multiple drugs concomitantly, as well as in validating other diagnostic tests [127]. The pilot study published in 2013 by Madrigal-Burgaleta et al. evaluated the use of DPTs for chemotherapeutic agents, its findings being further validated in two followings larger studies [121,127,128]. Ureña-Tavera et al. reported on the utility of DPTs when multiple drugs are associated [51]. Its usefulness has also been proved in evaluating the degree of tolerance to cross-reactive alternative drugs (e.g., possible chemotherapeutic options from the platinum salts group) [34]. There are, however, significant risks (some severe, corresponding to Brown’s classification) associated with a procedure lacking general standardized protocols for the majority of drugs [129,130]. Nevertheless, when DPT is repeatedly performed before the rapid drug desensitization (RDD) procedures, it could prevent such unnecessary therapeutic measures in non-hypersensitive patients, as is shown in the Ramon y Cajal University Hospital study (where 33% to 56% of the subjects included, depending on the substance tested, had a negative DPT could bypass RDD [127]). RDD represents a treatment option for patients in the case of hypersensitivity to the first line of medication, protecting against the risk of anaphylaxis. In vitro mast cell models of IgE antigen desensitization have provided the necessary therapeutic protocols to protect these highly sensitized patients. RDD represents an acceptable treatment option in specialized patient care, as well as an increased-risk therapeutic approach because the probability of an anaphylactic reaction is counterbalanced by the possibility of increasing the quality of life and life expectancy [131]. Although the optimal strategies for a systematic DPT implementation in the evaluation of chemotherapy-associated HSR remains a matter of discussion and subject to many variations, two elements are essential: an attentive patient selection and an adapted strategy in the management of risks related to the procedures [6]. The patients with an indication for this diagnostic test are the ones with immediate reactions (including anaphylaxis of milder degree with a favorable risk evaluation), as well as the individuals with delayed manifestations (other than severe cutaneous adverse reactions). On the other hand, the contraindications comprise a deficiency in proper risk management resources and the idleness of such a diagnostic test (when the patient is scheduled to switch to an alternate chemotherapeutic agent) [121,125,127].

#### 3.2.3. In Vitro Tests

##### Preclinical Stages of Drug Development

Despite that toxicity studies are irrelevant to predict the potential for sensitization during the preclinical development of various medicinal products, in vivo or in vitro tests have significantly gained importance; however, no in vivo or in vitro tests have been validated [132,133,134,135]. A point of reference in that respect is the OECD Test Guideline specifying in vitro test methods relating to mechanisms included as key biological events pertaining to the Adverse Outcome Pathway [136]. The tests in question are the direct peptide reactivity assay (DPRA), the ARE-Nrf2 luciferase KeratinoSensTM assay, and the human cell line activation test (hCLAT), based on the differentiation operated by the United Nations Globally Harmonized System of Classification and Labeling of Chemicals between skin sensitizers and nonsensitizers [136,137,138,139,140]. The assays were developed on the underlying principle of maintaining the production of antigen-specific T cells during the sensitization phase. The extrapolation from the data suggested a potentially similar mechanism of action between medicines and chemical allergenic agents leads to the relevant use of these in vitro assays to determine the likelihood of hypersensitivity reactions during the preclinical stage of drug development.

##### Clinical Stages of Drug Development

Immediate HSRs are mediated by basophils upon specific recognition of allergens by surface IgE, when they release potent mediators accompanied by mechanistically distinct surface expression of activation markers CD63 and CD203c. The level of IgE-mediated activation can be revealed by in vitro studies by flow cytometry after staining the cells with panels of specific fluorochrome-antibody conjugates. DURAClone is one such kit containing dry antibodies against CD3, CD45, CD63, CD203c and CD294 [141,142,143].

Diagnostic tools such as specific IgE (sIgE) or total IgE (tIgE) determination, basophil activation test (BAT), and tryptase determination have been developed to further evaluate chemotherapy-related HRS [6]. SIgE is a widely applied technique when evaluating patients’ reactions to platinum compounds. The prospective study conducted by Alvarez-Cuesta et al. in 2015 proved that a positive ST, as well as a sIgE for oxaliplatin, are proper techniques in confirming hypersensitivity for the chemotherapeutic agent. However, a negative result has provided less usefulness [6,121]. In contrast, there has been only one report of sIgE used for taxanes [144]. The capacity of tIgE to predict platins’ associated HSR has been demonstrated in the studies performed by Caiado et al. and Madrigal-Burgaleta et al. [127,145]. BAT has had limited use in diagnosing HSR induced by chemotherapy, more specifically related to platins [146,147,148]. Tryptase determination is an anaphylaxis biomarker for IgE-dependent and independent reactions, more useful when measured in dynamic (baseline versus time-of-reaction level). It can evaluate the initial HSR, as well as a positive DPT or a reactive RDD [149,150].

The lymphocyte transformation test (LTT) evaluates the degree of in vitro T cells’ proliferation as a response to a drug, permitting the deduction of a previous in vivo reaction due to sensitization. The major advantage of this technique is the possibility of applying it to many therapeutic agents, because drug-specific T cells are involved in almost all therapeutic agent hypersensitivity reactions. However, an in vitro drug-related T cell proliferation is difficult to implement in a clinical setting, the test in its essence being cumbersome and technically demanding [151].

## 4. Conclusions and Perspectives

Despite recent advancements in cancer patient care, systemic chemotherapy remains the backbone of oncological treatments and HSRs to the administrated anticancer drugs are a frequent side effect. These infusion reactions are unpredictable, non-dose related, and unexplained by the drug cytotoxicity, and can have potentially lethal consequences. More, these HSRs could endanger proper patient treatment, therefore the correct diagnosis and efficient management of oncologic patients presenting HSRs are crucial to not deprive patients of first-line treatment. 

Based on the interval between drug exposure and the onset of HSRs clinical manifestations, these reactions can be classified as immediate and non-immediate. Immediate HSRs occur during the drug administration process or within the first hours after treatment and are frequently encountered in oncological treatments, urticaria, angioedema, rhinoconjunctivitis, bronchospasm, and anaphylaxis being signature manifestations of immediate HSRs [152]. 

Although not as frequent and not life-threatening, leading to mild-to-moderate symptoms, delayed HSRs remain a safety concern of interest because of their incidence, which is not negligible according to patient reporting and possibly underestimated to elicit more extensive research. Therefore, the pathophysiology of such delayed reactions remains largely unclear or agent-specific, not associated with any chemotherapy agent(s)/class; in addition, their very nature as drug-induced or as a biological process has remained undetermined [153,154]. 

Keeping in mind the impact of HSRs on the patients, more in-depth research and in vitro studies are needed in preclinical product development and further clinical use, aiming to determine such agents’ potential for delayed allergic reactions. Given that regular toxicity studies are not relevant to point to possible delayed hypersensitivity reactions triggered by systemic products and from the perspective of mechanisms involved in the early and late stages phases of hypersensitivity events, in vitro and in vivo tests remain the means to reveal the cells activated and the mediators released in this process [137,138]. More, a modern approach to cancer therapy relies on the use of nanocarriers such as liposomes or polymeric nanoparticles to deliver drugs to tumor cells [155]. Not only that the use of nanoshuttles to deliver chemotherapeutic agents to cancer cells present numerous advantages compared to traditional therapy (e.g., drug payload protection, drugs bioavailability enhancement, tumor specificity improvement), but also can protect blood cells components from chemotherapy-induced cytotoxicity [156], being, therefore, a strategy that can be further explored for overcoming chemotherapy-induced HSRs.

## Figures and Tables

**Table 1:** Gell and Coombs classification of hypersensitivity reactions.

Type I	IgE antibody-mediated reactions, e.g., anaphylaxis
Type II	Antibody-mediated cytotoxic reactions, e.g., hemolytic anemia, thrombocytopenia, blood transfusion reactions
Type III	Immune complex-mediated hypersensitivity, e.g., serum sickness, vasculitis
Type IV	Delayed T cell-mediated responses, e.g., allergic contact dermatitis, maculopapular exanthema, erythema multiforme, toxic epidermal necrolysis, Drug-induced hypersensitivity syndrome (DiHS), hypersensitivity pneumonitis

**Table 2:** Classes of antineoplastic therapy with potential hypersensitivity reactions [3,8,9,10,11,12,13,14,15,16,17,18].

CLASS	Representatives	Examples	Administration Route	Indications	Hypersensitivity	Adverse Reactions	
						Cutaneous	Systemic
ALKYLATING AGENTS [10,11]	Nitrogen mustard derivatives	Cyclophosphamide	Oral, IV	Hodgkin or non-Hodgkin acute and chronic lymphatic or myeloid leukemia, malignant lymphomas, OC, BC, LC, neuroblastoma	Anaphylaxis	Urticaria, angioedema, acral erythema, erythema multiformae, nail disorders, stomatitis, vasculitis, SJS, TEN, RRR, pruritus	Pulmonary toxicity, BMS hemorrhagic cystitis, HVOD, EFT, cardiac toxicity, UTRT
		Melphalan	oral	polycythemia vera, multiple myeloma, advanced breast carcinoma, breast adenocarcinoma	Anaphylaxis	Rash, urticaria, angioedema, pruritus, stomatitis, scleroderma, erythema, alopecia	Interstitial pneumonitis pulmonary fibrosis, BMS
		Mechlorethamine	IV	CML, CLL, CTCL, HL, NHL, brain	Anaphylaxis	Urticaria, angioedema ACD, hyper pigmentation, pruritus, erythema multiformae, SJS	Lymphocytopenia
	Triazines	Dacarbazine	IV	malignant melanoma, sarcoma, islet cells, PC, neuroblastoma	Anaphylaxis	Rash, photosensitivity, ISR, urticarial	fever, hypereosinophilia, allergic hepatitis, BMS
		Temozolomide	oral	brain cancer, melanoma	Anaphylaxis	Rash, pruritus, photosensitivity, acral erythema, urticaria, pigmentation, MPR, SJS, TEN, alopecia	Fever, peripheral edema, pneumonitis, thrombocytopenia, neutropenia
	Ethylenimines	ThioTEPA	IV, intrathecal, intravesicular	OC, BC, bladder cancer leukemia, multiple myeloma	Anaphylaxis	Rash, urticaria, angioedema, pruritus, pigmentation, ISR, alopecia, dermatitis	Fever, wheezing, BMS, thrombocytopenia, anemia, leukopenia
ANTIMETABOLITES [10,11]	Folate antagonists	Methotrexate	Oral, IV	gestational choriocarcinoma, choriocarcinoma and hydatidiform mole, BC, head, and neck cancer, LC (scaly and with small cells), NHL	Anaphylaxis	Acral erythema, rash, pruritis, urticaria, vasculitis, erythema multiformae, photosensitivity, stomatitis, psoriasis, pigmentation, SJS, TEN, alopecia	Bronchospasm, pulmonary infiltrates, hemolytic anemia, agranulocytosis, myelosuppression, hepatotoxicity
		Pemetrexed	IV	malignant pleural mesothelioma, NSCLC	-	Rash, pruritus, vasculitis, mucositis, stomatitis, TEN, alopecia	Neutropenia, thrombocytopenia, anemia, interstitial pneumonitis, dyspnea, mucositis
	Pyrimidine analogues	Capecitabine	oral	metastatic CRC	-	Acral erythema, erythema, exfoliative dermatitis, PPK, stomatitis, photosensitivity, hyperpigmentation, RRR, IRAK	Lymphopenia, thrombocytopenia, neutropenia, anemia
		Cytarabine	IV	acute nonlymphocyticleukemia, ALL, chronic myelocytic leukemia	Anaphylaxis	Urticaria, angioedema, acral erythema, pruritus, TEN, vasculitis, AGEP, NEH, MPR	Thrombocytopenia, leucopenia, fever, dyspnea, ARDS, hepatotoxicity, bleeding
		5′-Fluorouracil	IV	CRC, BC, GC, PC, liver, uterine, OC, bladder cancer	Anaphylaxis	Acral erythema, MPR, photosensitivity, stomatitis, contact dermatitis, ISR, alopecia	Myelosuppression, mucositis
		Gemcitabine	IV	NSCLC, PC, OC, BC, bladder cancer	-	Cutaneous: Pruritus, MPR, acral erythema, bullous dermatitis, vasculitis, stomatitis, SJS, TEN, RRR,	Fever, pneumonitis, leukopenia, neutropenia, anemia, thrombocytopenia
PLATINUM COMPOUNDS [17]	DNA cross-linkers	Carboplatin	IV	OC, LC, BC, head and neck, testicular, brain (children) cancers	Anaphylaxis	Rash, pruritus, urticaria, angioedema, erythema, edema	BMS, bronchospasm, dyspnea, peripheral neuropathy
		Cisplatin	IV	sarcomas, OC, lymphomas, LC, germ cell cancers	Anaphylaxis	flushing, rash, urticaria, pruritus	Bronchospasm, dyspnea, hemolytic anemia, renal toxicity
		Oxaliplatin	IV	Metastatic CRC, OC, GC	Anaphylaxis	Flushing, erythema, urticaria, angioedema, pruritus, rash, acralerythema, RRR	Fever, dyspnea, wheezing, types II and III hypersensitivity
MITOTIC INHIBITORS [3]	Vinca alkaloids	(a) Vinblastine (b) Vincristine (c) Vindesine (d) Vinorelbine	IV	(a) HL, NSCLC, head and neck, NHL, BC, Kaposi syndrome. (b) HL, WT, leukemia, Ewing sarcoma, rhabdomyosarcoma, neuroblastoma, nephroblastoma, embrionary child tumors, osteosarcoma. (c) Melanoma, LC, BC, leukemia. (d) BC, bone, NSCLC,	Anaphylaxis	Acral erythema, rash, phlebitis, cellulitis, stomatitis, nail lesions, alopecia, Raynaud’s phenomenon	Fever, bronchospasm, ARF, pulmonary edema, pleural effusion, interstitial pneumonitis
	Taxanes	Docetaxel	IV	OC, BC, LC, AIDS-related Kaposi sarcoma	-	Acral erythema, scleroderma-like, CLE, stomatitis, RRR, ISR, nail abnormalities, TEN	Anemia, neutropenia, leukopenia, bronchospasm, dyspnea, back pain
		Paclitaxel	IV	BC, NSCLC, metastatic hormone-resistant prostate cancer, GC, squamous cell of head and neck cancers	-	Acral erythema, ISR, erythema multiformae, RRR, AGEP-like, SIS, alopecia	Neutropenia, BMS, hypersens pneumonitis, dyspnea, back pain
		Cabazitaxel	IV	hormone-resistant metastatic prostate cancer	-	Rash	Myelosuppression, nausea, vomiting, constipation, peripheral -neuropathy, neuromuscular pain
TOPOISOMERASE INHIBITORS [8]	Topoisomerase I inhibitors	Irinotecan	IV	CRC, squamous cell carcinoma of the cervix	Anaphylaxis	Rash, alopecia	Neutropenia, anemia, dyspnea
	Topoisomerase II inhibitors	Daunorubicin	IV	AML, ALL, neuroblastoma	Anaphylaxis	Rash, urticaria, angioedema, hyperpigmentation	BMS, fever, cardiac toxicity
		Doxorubicin	IV	HL, hematologic, OC, BC, LC, bladder cancer, Kaposi’s	Anaphylaxis,	Rash, acral erythema, ISR, pruritus, urticaria, angioedema, NEH, RRR	Myelosuppression, bronchospasm, cardiotoxicity
		Mitoxantrone	IV	BC, AML, NHL, ALL	Anaphylaxis	Rash, ISR, purpura, nail discoloration, stomatitis, alopecia	Myelosuppression,
DISRUPTION OF PROTEIN SYNTHESIS [12]		L-Asparaginase	IV	ALL, AML	Anaphylaxis	Urticaria, angioedema, rash, pruritus, stomatitis, TEN	Laryngospasm, BMS, pancreatitis
MONOCLONAL ANTIBODY [15]		Rituximab	IV	follicular lymphoma stage III-IV, diffuse big B cell NHL, LLC	Anaphylaxis	Paraneoplastic pemphigus, lichenoid dermatitis, vesiculobullous dermatitis, SJS, TEN	Pulmonary events, renal toxicity, neutropenia, serum sickness, fever, lymphopenia, chills, asthenia
		Brentuximab vedotin	IV	HL, Systemic anaplastic large cell lymphoma, Cutaneous T-cell lymphoma	Anaphylaxis	Rash, pruritus, SJS, alopecia	Cytopenia, immunogenicity, URTI, pyrexia, nausea, vomiting, fatigue, cough
		Cetuximab	IV	CRC, Squamous cell cancer of the head and neck	Anaphylaxis	Acneiform rash, nail changes, xeroderma, paronychial inflammation, pruritus	Electrolyte imbalance, infection, headache, diarrhea
		Trastuzumab			Anaphylaxis/Angioedema	Rash, nail disorders, pruritus	Neutropenia, anemia, thrombocytopenia, pulmonary events, LVD, chills, fever, URTI, headache, cough, stomatitis, mucosal inflammation
SIGNAL TRANSDUCTION INHIBITORS [13]	Non-receptor tyrosine kinase inhibitors	Bosutinib	IV	Ph+CML	Anaphylaxis	Rash, pruritus	Neutron, anemia, edema, hepatotoxic, pneumonia, pyrexia, cough, renal toxicity, EFT
	Receptor tyrosine kinase inhibitors	Lapatinib	IV	Metastatic BC	Anaphylaxis	Rash, HFSR, pruritus, xerosis, paronychia, nail disorders, alopecia	Cardiac toxicity, hepatotoxicity, ILD, diarrhea
HORMONES, HORMONE ANALOGS, AND HORMONE ANTAGONISTS [18]	Aromatase inhibitors	Exemestane	oral	BC in Postmenopausal women	Anaphylaxis	Rash, urticarial, pruritus, cutaneous vasculitis, erythema multiformae, AGEP, alopecia, dermatitis	Hot flushes, arthralgia, dyspnea, decreased bone density, EFT
		Letrozole	oral	BC in Postmenopausal women	Anaphylaxis	Angioedema, rash, erythema multiformae, TEN, alopecia	Flushing, dyspnea, EFT, diaphoresis, arthralgia, hypertension, peripheral edema, decreased bone density
	Gonadotropin-releasing hormone analogs (GnRH agonists)	Goserelin	IV	Prostate cancer, BC	Anaphylaxis	Rash, itching, RPCS, acne, seborrhea, alopecia	Hot flushes, DIDF, anemia, osteoporosis, vaginitis
		Leuprolide		Prostate cancer, BC	Anaphylaxis	Rash, injection site granuloma, pruritus, xerosis, ecchymosis, photosens, pigmentation	Hot fushes, DIDF, thrombo, anemia, peripheral edema

ACD = allergic contact dermatitis, AGEP = acute generalized exanthematous pustulosis, ALL = acute lymphocytic leukemia, AML = acute myeloid leukemia, ARDS = acute (adult) respiratory distress syndrome, ARF = acute respiratory failure, BC = breast cancer, BMS = bone marrow suppression, CLL = chronic lymphocytic leukemia, CML = chronic myeloid leukemia, CML = chronic myeloid leukemia, CRC = colorectal cancer, CTCL = cutaneous T cell lymphoma, DIDF = drug-induced disease fare (tumor fare effect), EFT = embryo-fetal toxicity, GC = gastric cancer, HER2 human epidermal growth factor receptor 2, also known as HER2/neu, ErbB2, CD340, p185 or EGFR2, HFSR = hand-foot skin reaction, HL = Hodgkin’s lymphoma, HVOD = hepatic veno-occlusive disease, ILD = interstitial lung disease, IRAK = inflammatory response in actinic keratosis, ISR = injection site reaction, IV = intra venous, LC = lung cancer, LVD = left ventricular dysfunction, MPR = maculopapular rash, MPR = maculopapular rash, NEH = neutrophilic eccrine hidradenitis, NHL = non-Hodgkin lymphoma, NSCLC = non-small-cell lung cancer, OC = ovarian cancer, PC = pancreatic cancer, Ph+ = Philadelphia chromosome-positive, PPK = palmar-plantar keratoderma, RPCS = relapsing polychondritis cutaneous symptoms, RRR = radiation recall reaction (dermatitis), SJS = Steven’s-Johnson syndrome, TEN = toxic epidermal necrolysis, URTI = upper respiratory tract infection, UTRT = urinary tract and renal toxicity, VEGFR2 vascular endothelial growth factor receptor 2, also known as KDR (kinase insert domain-containing receptor), WT = Wilms’ tumor.

**Table 3:** Risk factors associated with platinum agents HSR.

	Carboplatin [6,20,21,22,23,24,25,26,27]	Oxaliplatin [6,20,22,23,28,29,30,31]	Cisplatin [6,20,22]
Risk factors			
Previous multiple exposures	√	√	√
Previous treatment with another platinum drug	√	√	√/x
High cumulative dose	√	√	NA
Long platinum-free interval	√	√	NA
Positive ST result after a previous reaction	√	√	NA
Female gender	√	√	√
Young age	√/x	√/x	NA
Atopy history	√/x	√/x	NA
Cardiovascular involvement	√/x	√/x	NA
Others	Certain chemotherapy regimens (higher with carboplatin/paclitaxel)	Palliative second-line setting	Concomitant radiation
	BRCA 1/2 mutation		
	Weekly infusions in children with high-grade glioma		

BRCA = Breast cancer gene. √-proved as a risk factor for HSR; x-not proven as a risk factor for HSR; NA = information not available.

**Table 4:** Types of reactions reported for each drug.

	Immune-Mediated Reactions		Pseudo-Anaphylactic Reactions	Substance Responsible for HSR	Observations	References
	Immediate	Delayed				
Platinum analogs					Ig-E mediated in most cases. Direct mast cell degranulation in other cases.	
Cisplatin	√	x	x	Chimiotherapeutic agent		[32,33,34]
Carboplatin	√	√	√	Chimiotherapeutic agent		[21,22,26,36,37]
Oxaliplatin	√	√	x	Chimiotherapeutic agent		[17,23,28,41,43,46]
Taxanes					Direct mast cell degranulation in most cases. IgE sometimes reported.	
Paclitaxel	√	√	√	Both chemotherapeutic agent and solvent (ChremophorEl)		[6,7,20,55,57]
Docetaxel	√(rare)	√	√	Both chemotherapeutic agent and solvent (Polysorbate 80)		[58,61,62,63,64]
Cabazitaxel	x	x	√	Both chemotherapeutic agent and solvent (Polysorbate 80)		[69,70,71,72]
Nab-paclitaxel	√	x	x	NK		[73,76,78]
L-asparaginase	√	√	√	*Mostly induced by E. coli*-derived asparaginase *Erwinia* 332 asparaginase	Mostly specific anti-drug antibodies	[6,7,79]
Bleomycin	√(rare)	√	√(rare)	NK	Presumably direct mast cell activation	[83,84,85]
*Topoisomerase inhibitors*						
Etoposide/teniposide	x	x	√	Solvent polysorbate-80	Direct mast cell activation	[6,87,88]
Doxorubicin	√(rare)	x	x	NK		
pegylated liposomal doxorubicin	x	x	√	surface component of the liposome	Direct complement activation	[7,91,94]
Alkylating agents						
cyclophosphamide	√(rare)	x	x	metabolites phosphoramide mustard, and acrolein	Presumed IgE-mediated reactions to the two main metabolites	[95,96,97]
ifosfamide	√(rare)	x	x	most ifosfamide-related reactions are considered to be due to MESNA		[98,99,100]
Antimetabolites						
Citarabine	√(rare)	√(rare)	√	NK	Infusion reactions are usually described as “flu-like cytarabine syndrome”	[101,102]
Monoclonal antibodies					most of the infusion reactions associated coincide with the cytokine-mediated pattern/IgE-mediated reactions also described	
Rituximab	√(rare)	√(rare)	√	NK		[103,104,105,106]
Trastuzumab	√(rare)	x	√	NK		[111,112,113]
Cetuximab	√	x	√	galactose-alpha-1,3-galactose, oligosaccharide presents on the cetuximab mouse-derived heavy chain		[115,116,117]

√-Yes; x-No.

**Table 5:** Non-irritating concentrations of chemotherapeutic drugs for STs (adapted after [6]).

Drug	Prick Test Dilutions (mg/mL)	Intradermal Test Dilutions (mg/mL)
Carboplatin	1/1 (10)	1/100 (0.1)
		1/10 (1)
Cisplatin	1/1 (10)	1/100 (0.01)
		1/10 (0.1)
		1/1 (1)
Oxaliplatin	1/1 (5)	1/100 (0.05)
		1/10 (0.5)
		1/1 (5)
Paclitaxel	1/10 1 (6)	1/1000 (0.001 [0.006])
		1/100 (0.001 [0.006])
		1/10 (0.6)
Docetaxel	1/1 4 (1)	1/100 (0.04 [0.01])
		1/10 0.4 [0.1]
Procarbazine	1/1 (5)	1/100 (0.05)
Gemcitabine	1/1 (38)	1/1000 (0.0038)
		1/100 (0.038)
		1/10 and 1/1
Methotrexate	1/1 (10)	1/100 (0.1)
		1/10 (1)
		1/1 (10)
L-Asparaginase	A drop of reconstitute 5000 KU	0.01 mL of reconstitute 5000 KU
